# Evaluation of the Impact of Optical Coherence Tomography on Pediatrician Otologic Examination Judgment

**DOI:** 10.1002/oto2.41

**Published:** 2023-03-01

**Authors:** Malinda S. Teague, Ryan M. Nolan

**Affiliations:** ^1^ Health of Women, Children, & Families Division Duke University School of Nursing Durham North Carolina USA; ^2^ PhotoniCare, Inc. Champaign Illinois USA

**Keywords:** middle ear effusion, optical coherence tomography, otitis media, otoscopy, pediatrics

## Abstract

Accurate diagnosis of otitis media is imperative to judicious antibiotic prescription. Visualization of the tympanic membrane and accurate identification of middle ear effusion with standard otoscopy is inherently challenging in pediatrics, especially in the youngest children who are most at risk for otitis media. With the average diagnostic accuracy among primary care physicians of 50% and accurate identification of normal tympanic membrane versus acute otitis media versus otitis media with effusion ranging from 30% to 84% among pediatricians, there is great opportunity for diagnostic improvement and decreasing unnecessary antibiotic use. In a 96‐pediatrician‐blinded otoscopy diagnosis quiz, addition of optical coherence tomography, a novel depth‐imaging technology, resulted in a 32% improvement in fluid identification, and 21% increase in diagnostic accuracy. This study suggests that the clinical use of this technology promises to improve diagnostic accuracy and antibiotic stewardship in pediatrics.

Next to the common cold, acute otitis media (AOM) is the most frequently diagnosed pediatric illness in the United States, accounting for 1‐in‐9 primary care office encounters.^1^ Over 70% of children experience ≥1 episode of AOM by age 1 year^2^, ^3^ and 93% by age 7 years.^2^ Ear infections are accompanied by middle ear effusion (MEE). In otitis media with effusion (OME), the clear, watery or mucoid MEE present does not warrant antibiotics.^4^ AOM includes purulent MEE, represents an infectious process, and is the most cited pediatric indication for antibiotics.^4^ Approximately 20% of children have recurrent AOM, often resulting in surgical tympanostomy tube placement (TTP). TTP ensues in 6.26 million (8.6%) US children,^5^ totaling ~$4 B in healthcare expenses.

Current primary care tools include the ubiquitous otoscope to subjectively assess tympanic membrane (TM) visual properties along with AOM‐associated symptoms. Average diagnostic accuracy among primary care physicians is ~50%,^6^, ^7^ specifically ~30% to 84% for pediatricians.^7^, ^8^, ^9^, ^10^, ^11^, ^12^, ^13^, ^14^, ^15^ This leads to consistent over‐diagnosing of AOM 7% to 53% (mean = 27%) of the time,^6^ resulting in unnecessary antibiotics ~29% of the time.^8^ Though more accurate, more advanced examination techniques go largely unused in primary care (38% pneumatic otoscopy reported use; 7% tympanometry).^16^, ^17^

American Academy of Otolaryngology–Head and Neck Surgery/American Academy of Pediatrics guidelines advocate for new/improved technologies for objectively diagnosing MEE.^1^, ^4^ Optical coherence tomography (OCT) is a well‐established, noninvasive imaging modality with >150 publications on OCT use to visualize the TM and middle ear for relevant pathology.^18^, ^19^, ^20^ Considered the optical analogue of ultrasound, OCT uses near‐infrared light to produce depth‐resolved images with micron‐scale resolution. This study's primary objective is to evaluate OCT's impact on specifically pediatrician otologic examination judgment.

## Methods

A blinded reader image quiz was conducted with 96 clinician volunteers via website using tablets at AAP Experience 2019 National Conference. Brief training was provided on understanding/interpreting OCT images. Readers were first instructed to identify OCT signal from the TM, primarily seen as a white ribbon (Figure 1.1). Then readers compared/analyzed the space immediately above versus below that TM ribbon for differences in signal intensity/brightness and density, specifically on the middle ear side, which indicates the presence of MEE. A representative image dataset was presented illustrating that OCT signal from any MEE present can vary depending upon MEE contents.

**Figure 1:**
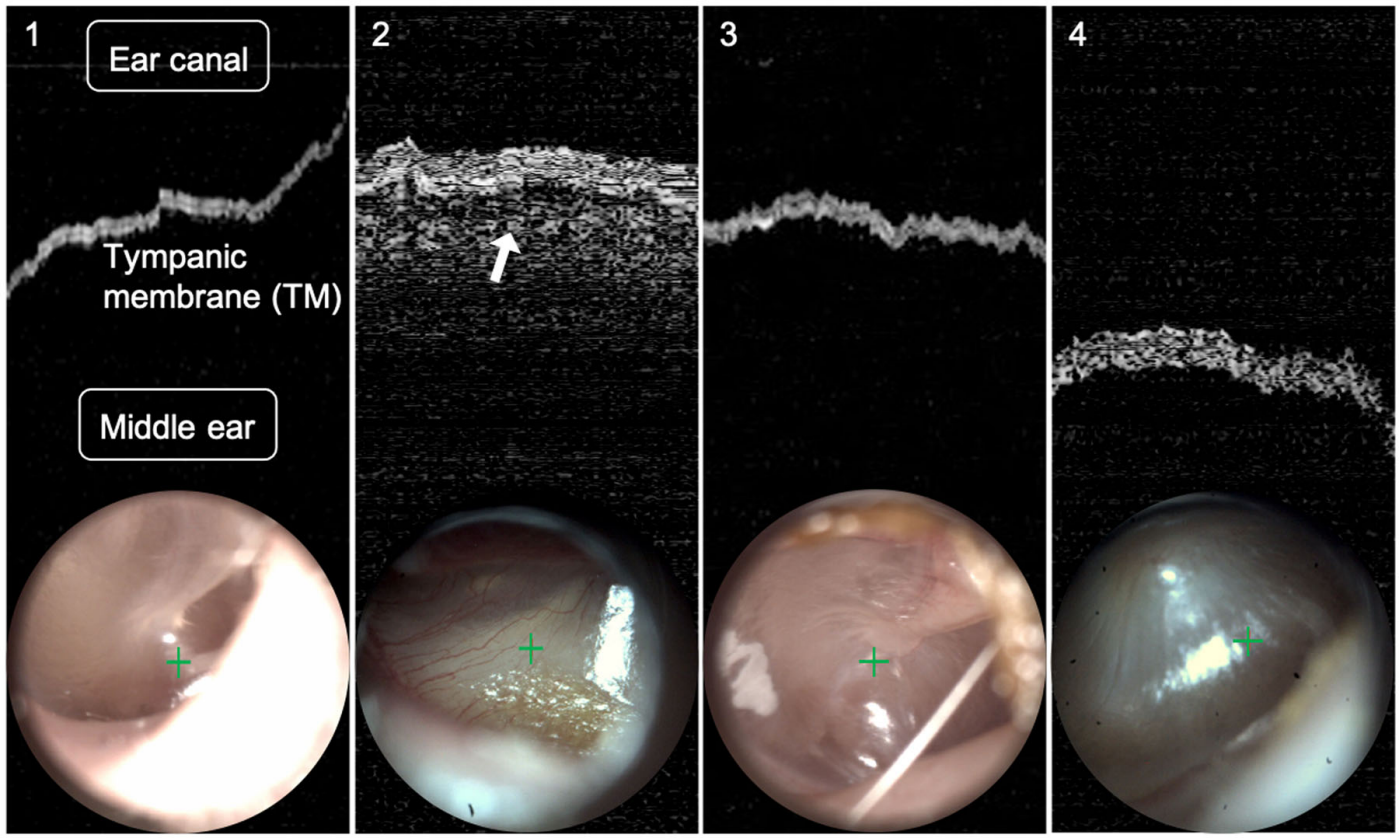
Four pediatric ear datasets used in the blinded reader quiz. (1) Representative normal ear; no MEE. (2) Representative AOM; MEE indicated by arrow. (3, 4) Two “borderline” cases representing the range between obviously normal versu. AOM ears. AOM, acute otitis media; MEE, middle ear effusion.

Heterogeneous, low‐brightness scattering OCT signal is seen in OME due to sparse MEE contents in clear, serous effusions. Purulent MEE (ie, AOM) presents as homogeneous, high‐brightness scattering OCT signal due to increased turbidity from dense effusion contents (eg, bacteria, immune cells, pus). Though there is general aggregation of these OCT image qualities, there is a continuum of the contents present in MEEs; resulting OCT scattering ranges between the above descriptions depending upon the amount/type of MEE contents.^18^, ^19^, ^20^

The quiz included 4 pediatric case studies, first with patient history and otoscopy image:

- Case 1: Representative normal ear
- Case 2: Representative AOM with pronounced erythema and purulent MEE visible in otoscopy and OCT
- Cases 3 to 4: Representative “borderline” cases on the spectrum between normal and AOM

Blinded readers evaluated for evidence of MEE, diagnosed Normal versus AOM versus OME, then ranked their confidence from 1 to 5 (5 = highest confidence). The corresponding OCT image was then revealed, and readers were given the option/ability to change their assessments.

This study was determined to meet the definition of research not involving human subjects by retrospective review of the Duke University Health System IRB, approval #Pro00107958.

## Results

As shown in Table 1, readers showed improvement in diagnostic accuracy and confidence in all 4 cases following inclusion of OCT, specifically 32% improvement in MEE identification and 21% increase in diagnostic accuracy. The proportion of readers who changed their diagnosis post‐OCT was considerable for cases 1, 3, and 4. Case 2 resulted in 7% increased post‐OCT diagnostic accuracy, with 9% of readers changing their diagnosis despite a high initial diagnostic accuracy. Cases 1 to 3 post‐OCT diagnostic accuracies were between 81% and 98%, while Case 4 was 47%.

**Table 1:** OCT Impact by Case

	Case 1	Case 2	Case 3	Case 4
OCT impact on correct middle ear effusion identification				
Pre‐OCT	70 (73%)	87 (91%)	40 (42%)	21 (22%)
Post‐OCT	83 (86%)	94 (98%)	78 (81%)	45 (47%)
OCT impact on diagnosis change				
No. of diagnosis changes	29 (30%)	9 (9%)	46 (48%)	40 (42%)
OCT impact on confidence change				
No. of confidence increases	3 (33%)	12 (50%)	2 (25%)	8 (47%)
Amount of confidence change	0.6	0.4	0.2	0.5

Abbreviation: OCT, optical coherence tomography.

## Discussion

Pediatricians' ability to interpret OCT images of the middle ear in this study after only brief training is encouraging. The high diagnostic accuracy and improved diagnosis post‐OCT are consistent with prior literature, illustrating the promise for more accurately identifying MEE than current clinical tools.^18^ Case 4 presented the most difficulty, with a significant difference in post‐OCT diagnostic accuracy. This case was likely problematic due to the possibly confounding increased TM thickness in OCT compared to Cases 1 or 3. More robust training, including nuances of thickened TM OCT signal compared to the presence of MEE would be helpful.

Further quiz limitations include participant variable vested interest in completing the quiz attentively and restricted number of quiz cases to accommodate conference attendees' schedules. To address this in future work, more controlled blinded reader recruitment, training, and quiz execution will be leveraged, including a larger, more diverse set of quiz cases and inclusion of chronic OME (mucoid MEE) examples. Also, a single digital otoscopy frame may not be equivalent to viewing the TM from different angles during traditional otoscopy, so including video or multiple images may facilitate more clinically representative otoscopy assessment.

## Conclusion

OCT‐otoscopy provides additional objective data to evaluate MEE, and therefore may enable more accurate diagnosis of middle ear disease. Recent evidence shows no significant difference between medical management of AOM versus TTP in children 6 to 35 months when identified by validated otoscopists, so there is need for improved diagnostics like OCT‐otoscopy.^21^ Synergy with machine learning to facilitate provider interpretation regardless of experience level needs exploration. Still, this study's results support the promise of OCT‐otoscopy as a more accurate standard‐of‐care for pediatric patients, improving antibiotic stewardship, eliminating unnecessary referrals, and decreasing healthcare costs associated with OM.

## Author Contributions

**Malinda S. Teague**, lead Duke IRB approval, study data analysis, and presentation of research; **Ryan M. Nolan**, design, conduct, study data analysis, and presentation of research.

## Disclosures

### Competing interests

Malinda Teague discloses minor financial interests in PhotoniCare as a member of its Clinical Advisory Board. Ryan Nolan discloses financial interests in PhotoniCare as a co‐founder and employee.

### Funding source

PhotoniCare, Inc. funded this study. No public or not‐for‐profit funding was received. The authors received no individual financial support for the research, authorship, and publication of this article.
